# Short-term episodes of imposed fasting have a greater effect on young northern fur seals (*Callorhinus ursinus*) in summer than in winter

**DOI:** 10.1093/conphys/cou021

**Published:** 2014-06-06

**Authors:** David A. S. Rosen, Beth L. Volpov, Andrew W. Trites

**Affiliations:** 1 Marine Mammal Research Unit, Fisheries Centre, University of British Columbia, AERL 247, 2202 Main Mall, Vancouver, BC, Canada V6T 1Z4; 2 School of Life and Environmental Sciences, Deakin University, Burwood, Victoria 3125, Australia

**Keywords:** Fasting, metabolism, northern fur seals, nutrition

## Abstract

An unexpected shortage of food may affect wildlife in a different way depending on the time of year when it occurs. We imposed 48 h fasts on six female northern fur seals (*Callorhinus ursinus*; ages 6–24 months) to identify times of year when they might be particularly sensitive to interruptions in food supply. We monitored changes in their resting metabolic rates and their metabolic response to thermal challenges, and also examined potential bioenergetic causes for seasonal differences in body mass loss. The pre-fast metabolism of the fur seals while in ambient air or submerged in water at 4°C was higher during summer (June to Sepember) than winter (November to March), and submergence did not significantly increase metabolism, indicating a lack of additional thermoregulatory costs. There was no evidence of metabolic depression following the fasting periods, nor did metabolism increase during the post-fast thermal challenge, suggesting that mass loss did not negatively impact thermoregulatory capacity. However, the fur seals lost mass at greater rates while fasting during the summer months, when metabolism is normally high to facilitate faster growth rates (which would ordinarily have been supported by higher food intake levels). Our findings suggest that summer is a more critical time of year than winter for young northern fur seals to obtain adequate nutrition.

## Abstract (precis)

Imposed 48-hr periods of fasting in six young (6 to 24 months) female northern fur seals did not decrease metabolism or increase thermoregulatory costs, but were more detrimental in the summer than the winter when higher metabolic costs normally required to fuel seasonal growth led to higher rates of mass loss.

## Introduction

Wild animals rarely have the benefit of a steady supply of dependable food resources. Natural variation in prey availability and foraging success, digestive constraints and life-history considerations (such as periods required for nursing or mating) that may preclude timely foraging will result in intermittent nutritional intake. Animals have a number of adaptations, including down-regulation of resting metabolism, decreased activity, alteration of digestive processes and increased levels of subsequent food intake, that can be employed to compensate for inconsistent prey intake encountered on a normal basis to maintain an optimal nutritional plane that maximizes growth and survival. However, these adjustments are intrinsically limited in scope (Rosen *et al.*, 2007). Furthermore, they may be over-taxed or not fully implemented during periods of unexpected food restriction experienced in less typical conditions (Rosen and Trites, 2002). The impact of unexpected periods of food restriction or fasting is likely to be greater for younger animals, given their relatively higher energetic requirements and their physical under-development and behavioural naïveté. Periods of unexpected short-term fasts may impact immediate survival due to negative energy balance or impact future reproductive success through decreases in body size (Wilson and Osbourn, 1960).

Northern fur seals (*Callorhinus ursinus*) are an example of a species where young animals may be challenged to acquire sufficient prey even in normal circumstances. These fur seals become nutritionally independent in November at ∼4 months of age, when they become largely pelagic entities (Ragen *et al.*, 1995). During this initial period of independence, they have little experience with deep-water foraging and their physical diving capacity is under-developed (Baker and Donohue, 2000; Shero *et al.*, 2012). It is likely that some northern fur seals have difficulty finding sufficient prey every day and, therefore, endure occasional fasts during their first months at sea. Mortality rates of northern fur seals are greatest during the at-sea phase of their first 2 years of their life (Lander, 1982; Trites, 1989). While predation rates may be substantial, the greatest source of natural mortality is likely to be an inability to catch sufficient prey to meet their energetic requirements.

These normal patterns of periodic under-nutrition may be exaggerated due to changes in the fur seals' biotic or abiotic environment; factors that have been implicated in the observed decline in fur seal populations (Trites, 1992; National Marine Fisheries Service, 2007). This population decrease is most notable on the Pribilof Islands in the Bering Sea, with a 6% yearly decline in the main breeding site at St Paul Island, Alaska (National Marine Fisheries Service, 2007).

Young foraging northern fur seals must contend with many potential bioenergetic handicaps to their survival, including higher mass-specific energy requirements than adults (York, 1994; National Marine Fisheries Service, 2007) and the fact that they may normally be operating at close to their maximal digestive capacity (Rosen *et al.*, 2012). Thermally, the fur seals' lower lipid stores (at least compared with phocid seals; Liwanag *et al.*, 2012) and high surface-to-volume ratios may encourage greater rates of heat loss. The thermal neutral zone of young northern fur seals has been demonstrated to be substantially narrower than that of more mature individuals (Dalton *et al.*,2014; Rosen and Trites, 2014). If young fur seals have trouble acquiring sufficient prey, potential heat loss may be magnified as they use remaining hypodermal lipid reserves to meet energy deficits (Castellini and Rea, 1992; Donohue *et al.*, 2000), which can result in a lethal spiral of escalating thermal costs (Hoopes, 2007; Rosen *et al.*, 2007).

Metabolic depression (the down-regulation of metabolic rates) is often cited as a common physiological adaptation to periodic food shortages among many species (Keys *et al.*, 1950). The theory is that decreasing required metabolic costs (including resting metabolic rate) will limit rates of mass loss due to insufficient energy intake (Guppy and Withers, 1999). Changes in the resting metabolic rate of young pinnipeds have been examined over periods of natural fasting of otariid pups during the nursing period (Rea *et al.*, 2000; Arnould *et al.*, 2001; Beauplet *et al.*, 2003; Verrier *et al.*, 2009) or during the brief post-nursing moult period (Donohue *et al.*, 2000), although the latter has been most intensely studied among phocid seal pups (Worthy and Lavigne, 1987; Nordøy *et al.*, 1990, 1993; Reilly, 1991; Rea and Costa, 1992; Lydersen *et al.*, 1997; Houser and Costa, 2003). However, the metabolic response of mammals during life-history stages or seasons when food shortages normally occur may be different from the response during periods when food shortages are unexpected. Only one published study has examined changes in resting metabolism in response to episodes of experimental fasting in a young otariid outside of the normal nursing period (Rosen and Trites, 2002).

It must also be noted that the metabolic depression response does not occur in physiological isolation and must be viewed in light of competing bioenergetic priorities. It is unclear, for example, how this strategy would be implemented during a life-history stage that is also characterized by an increased priority for growth (with its related concurrent increase in metabolism) and high environmental thermal challenges. Past studies have confirmed that changes in metabolism in response to under-nutrition in young Steller sea lions (*Eumetopias jubatus*) are seasonal in nature (Rosen and Trites, 2002), which is likely to reflect different energetic priorities formulated in response to natural changes in food supplies or other bioenergetic variables. Like most subpolar species, the energetic requirements of northern fur seals appear to differ with the time of year (Rosen and Trites, 2010). Therefore, metabolic depression might potentially be more effective or more likely to be invoked as a strategy to deal with periodic food shortages during different seasons. The impact of changes in metabolism or body composition due to episodes of under-nutrition on thermoregulatory capacity or growth might also be seasonal in nature.

The goal of our study was to determine the bioenergetic consequences of imposed food interruptions on young northern fur seals. We examined the seasonal response of young northern fur seals to short periods of imposed fasting over their first 2 years post-moult. We monitored changes in resting metabolic rates between seasons and over the course of the imposed fasts and quantified the potential effect of fasts on the energetic cost of thermoregulation through cold-water thermal challenges. We also measured seasonal differences in lipid stores and body mass and determined whether the rate of mass loss during the fast was related to aspects of seasonal energy budgets, such as the normal level of food intake or metabolic rate. Ultimately, we wanted to determine whether seasonal differences in energetic responses make young northern fur seals more susceptible to the effects of unexpected interruptions in food supplies at certain times of the year.

## Materials and methods

We tested the seasonal effects of food restrictions on six female northern fur seals between January 2009 and June 2010. The animals came from the Pribilof Islands post-weaning in October 2008, and were kept at the Marine Mammal Energetics and Nutrition Laboratory located in the Vancouver Aquarium (British Columbia, Canada) as part of a long-term captive research programme. The study employed longitudinal measurements on the six fur seals over the ages of 6–24 months old, during which they ranged in mass from 9.4 to 18.1 kg (overall mean ± SD 12.93 ± 2.0 kg). All experimental protocols were approved by the Animal Care Committees of the University of British Columbia and the Vancouver Aquarium.

The fur seals were normally fed previously frozen herring, supplemented with vitamins. Food intake levels were set by husbandry staff to maximize food intake while retaining their training capacity. For each set of trials, metabolism was measured as the rate of oxygen consumption immediately prior to and at the end of a 48 h fast, both in ambient air and while submerged in water at 4°C. The resting metabolic rate (RMR) of the fur seals was measured in a specially built chamber (340 l) via flow-through respirometry. Ambient air (3.7–18.5°C) was drawn through the chamber at 125 l min^−1^ using a 550-H Mass flow pump (Sable Systems, Las Vegas, NV, USA). A subsample of the excurrent air was desiccated via drierite and then passed through a Sable Systems CA-1B CO_2_ analyser and a FC-1B O_2_ analyser. Ambient air temperature and humidity were also recorded. Instantaneous oxygen and carbon dioxide concentrations were averaged every second, and recorded to a PC using Sable System's Expedata software. Raw instantaneous gas concentrations were converted to rates of oxygen consumption (, in millilitres of O_2_ per minute) using LabAnalyst X (M. Chappell, UC Riverside) that employed appropriate equations as given by Withers (1977).

For the pre-fast measures of metabolism, the animal had not eaten for 14–17 h. This period represented the animals' routine overnight fast and was probably long enough to avoid any metabolic effects of the heat increment of feeding and short enough to preclude any significant fasting effect. Post-fast measurements were made ∼48 h later, giving an approximate total fasting time of 65 h. For each set of measures, metabolism was first measured at ambient air temperatures while each animal was in a dry chamber for ∼25 min. The resting metabolic rate in dry conditions (RMRdry) was calculated as the average over the last 10 min of the sample. The chamber was then partly flooded (so that the torso of the fur seal was submerged, but not deep enough to permit swimming) with salt water from a large reservoir tank that maintained the water at 4°C. Water flowed continuously through the chamber (the water level was maintained via a physical overflow to prevent accidental flooding) to ensure a constant testing temperature. The chamber took ∼5 min to fill, and the fur seal remained in the chamber for a further 25 min. The resting metabolic rate in wet conditions (RMRwet) was calculated as the average during the last 10 min of this phase.

Body mass was determined at the start and end of the fast by having the fur seals position themselves on a platform scale (±0.01 kg). Body composition (specifically, percentage lipid mass) was estimated at the start (within 3 days) of the fast via deuterium dilution techniques (as detailed by Reilly and Fedak, 1990). Briefly, a background serum sample was obtained under veterinary-supervised isofluorane anaesthesia prior to an intramuscular injection of deuterated water at a measured dose of 0.10–0.15 ml kg^−1^. A second serum sample was obtained 100–120 min post-injection. Matched serum samples and appropriate dose samples were analysed by Metabolic Solutions (Nashua, NH, USA). These results were transformed into total body water and were then converted to total body lipid (in kilograms) using the ‘All animal’ equation in Table 5 of Arnould *et al.* (1996). The percentage body lipid was estimated by dividing estimated lipid mass by the known body mass.

Each of the six fur seals was tested seven times over the course of the 18 month experiment. Individual trial periods were opportunistic, and therefore, not distributed evenly over the experimental period. The timing and number of trials did not allow us to use the same four seasonal divisions as for other studies (Dalton *et al.*, 2014; Rosen and Trites, 2014). Instead, each trial was categorized *a priori* as either ‘winter’ (January 2009, March 2009, November 2009, March 2010) or ‘summer’ (July 2009, September 2009, June 2010). The separate assignment of the September and November trials was made partly on the basis of each being included with similar pre- and post-moult periods. Subsequent retesting found that the assignments of the September and November trials had no statistical impact on the final results.

Statistical differences in physiological parameters (body mass, metabolic rate and percentage lipid) attributable to season (winter or summer) or trial (pre-fast or post-fast) were determined via linear mixed-effects (LME) models in R 2.9.0 statistical software (R Core Team, Vienna, Austria). The testing environment (dry or wet) was also included as a potential fixed factor for testing differences between RMRdry and RMRwet. These models allow data from each animal within a trial and data from each animal across trial types to be treated as a repeated measure by considering the correlation between repeated measurements within and among animals (Pinheiro and Bates, 2000). Treating each animal as a random effect for all models permits inferences from the sample population to be applied to wild counterparts. The model tests for potential additive effects by comparing the predictive power of competing models that incorporate different combinations of significant factors. For each model comparison (including comparison with the null model that incorporates no fixed effects), the best-fit model was selected using a likelihood ratio test (LRT) as described by Pinheiro and Bates (2000) that selects the simplest model (least number of factors) that explains the greatest amount of variance.

Body mass is known to affect metabolism, although the exact nature of this relationship is the subject of considerable debate (e.g. Heusner, 1982; Glazier, 2005; Packard and Birchard, 2008). We therefore tested for changes or differences in metabolism in terms of absolute rates of oxygen consumption (in millilitres of O_2_ per minute), as well as including body mass as a covariate to account for simultaneous differences or changes in body mass. Although the potential effect of mass on metabolism was tested in this manner, we also report values in terms of mass-specific metabolism (in millilitres of O_2_ per minute per kilogram) for clarity and to facilitate comparisons with other published studies.

## Results

### Changes in body mass, body composition and food intake

**Figure 1:**
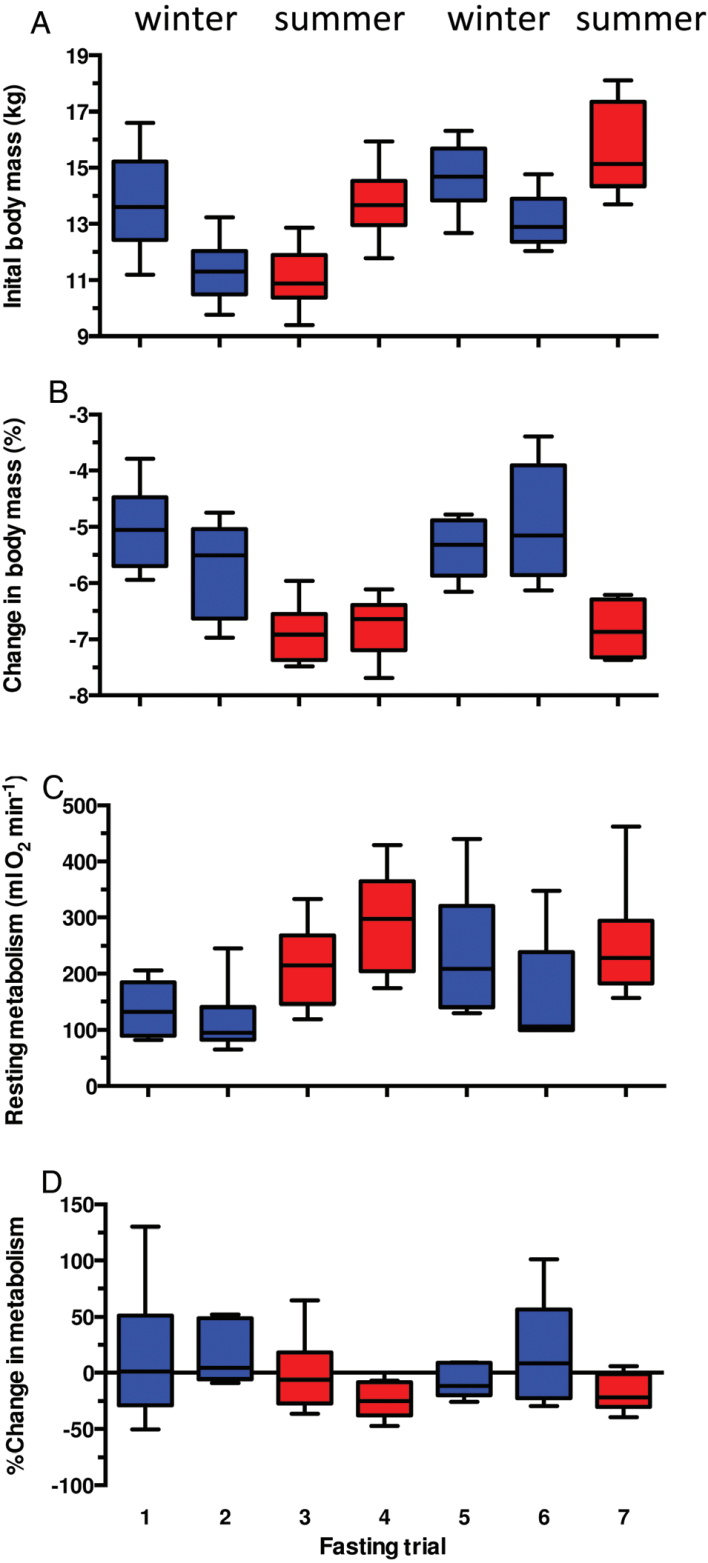
Experimental results for six female northern fur seals subjected to seven fasting trials. (**A**) Initial (pre-fasting) body mass (in kilograms). (**B**) Changes in body mass as a percentage of initial (pre-fasting) mass over each 65 h fast. (**C**) Initial (pre-fasting) rates of resting metabolism (in millilitres of O_2_ per minute) as measured in a dry metabolic chamber. (**D**) Percentage changes in resting metabolism during each dry trial. The fur seals were ∼6 months old during fasting trial 1, and 24 months old during fasting trial 7. Winter trials are designated by blue boxplots and summer trials by red boxplots. Boxplots illustrate the 25th–75th percentile bisected by the median, and whiskers designate the 95% confidence limits.

Developmental changes in body mass occurred over the 18 months it took to conduct the seven trials. The fur seals were at their lowest average body mass at the start of trial 3, when they were ∼6 months old (11.1 ± 1.1 kg ± SD), and greatest during the final trial when they were ∼24 months old (15.62 ± 1.7 kg; Fig. 1A). Non-linear growth meant that the average pre-fast body mass of the fur seals during the summer trials (13.5 ± 2.3 kg) was not different from that during the winter (13.2 ± 1.8 kg) at the start of the fasts (LRT = 0.16, *P* = 0.69). There was, however, a seasonal difference in the amount of mass lost over the course of the 48 h fast. The fur seals lost significantly more mass over the course of the fast during the summer trials (–0.92 ± 0.17 kg) than during the winter (–0.69 ± 0.10 kg; LRT = 22.41, *P* < 0.001). This seasonal difference was also apparent when mass loss was expressed as a percentage of initial body mass (summer = –6.8 ± 0.5%, winter = –5.3 ± 0.8%; LRT = 33.60, *P* < 0.001; Fig. 1B).

While average initial body mass did not differ between seasons, there was a seasonal difference in their initial estimated lipid stores. The fur seals possessed greater lipid stores during the winter compared with the summer, whether expressed as absolute lipid mass (3.4 ± 1.2  vs. 1.3 ± 0.6 kg; LRT = 23.52, *P* < 0.001) or as a percentage of total initial body mass (25.6 ± 7.0 vs. 9.8 ± 3.6%; LRT = 23.47, *P* < 0.001).

There were also seasonal differences in their gross energy food intake (LRT = 18.02, *P* < 0.001). Mean daily food intake during the 7 days prior to the fast was greater in the summer (1350 ± 14 g day^−1^) compared with the winter (971 ± 32 g day^−1^, *P* < 0.001). Given that the energy density of the food source was relatively constant throughout the study (6.9–7.6 kJ kg^−1^), this also meant that the fur seals had a significantly greater rate of energy consumption in the summer compared with the winter trials.

### Seasonal changes in metabolic rates

**Figure 2:**
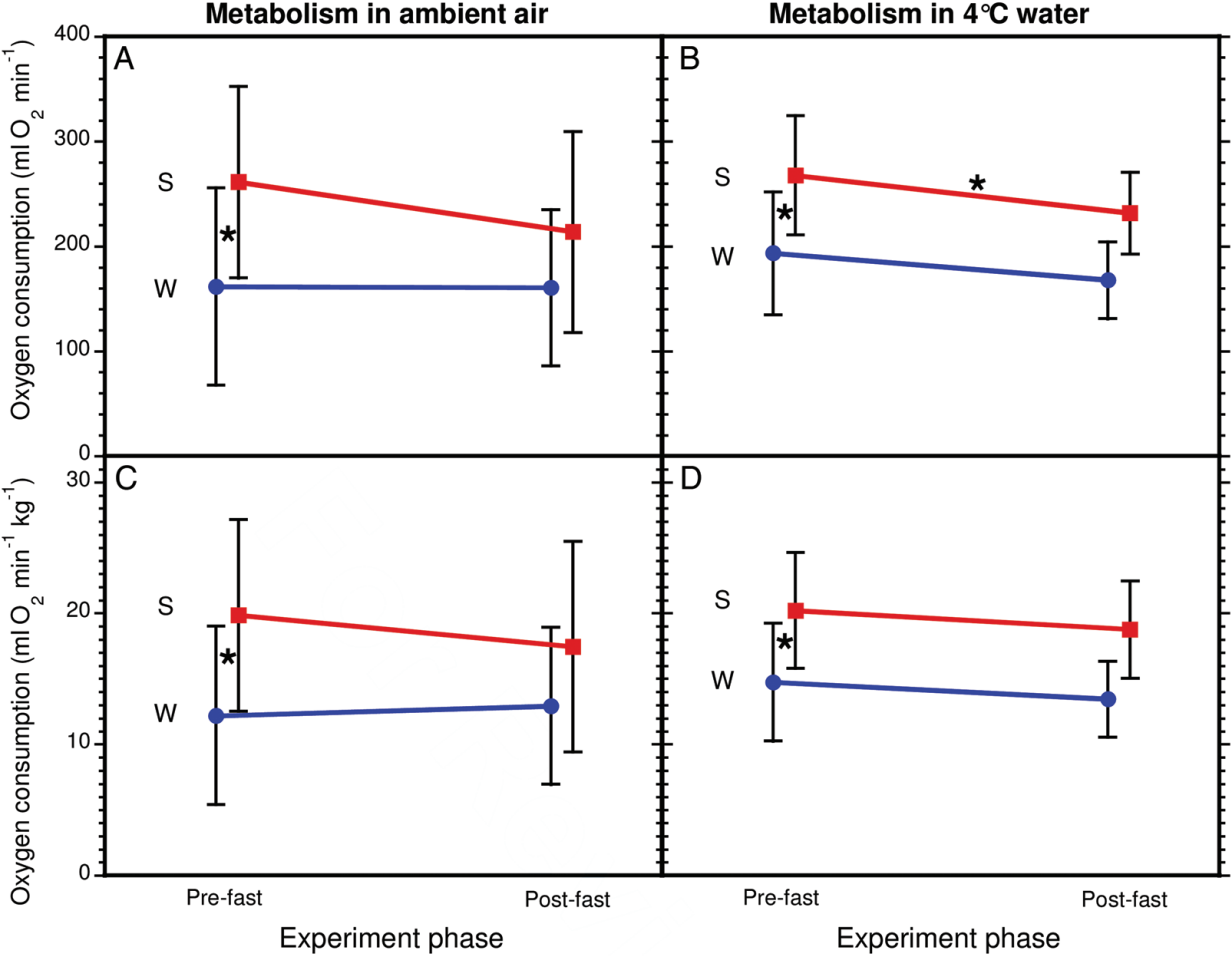
Changes in metabolism of six female northern fur seals over the course of 65 h fasts. Metabolism is presented as either absolute rates of oxygen consumption (**A** and **B**) or scaled to body mass (**C** and **D**). Measurements were made in ambient air (A and C) and when submerged in water at 4°C (B and D). Data are presented as means ± SD over the seven trials separated into either winter (‘W’, blue circles) or summer trials (‘S’, red squares). An asterisk indicates either statistically significant seasonal differences in pre-fast metabolic rates or significant differences between pre-fast and post-fast measures.

There was a significant seasonal difference in the initial (pre-fast) resting metabolic rates in ambient air (RMRdry), which were higher in the summer (261.3 ± 91.5 ml O_2_ min^−1^) compared with the winter (161.4 ± 94.0 ml O_2_ min^−1^; LRT = 39.00, *P* < 0.001; Figs 1C and 2A). Not surprisingly given the similar initial body mass of the fur seals in summer and winter, this seasonal difference in pre-fasting metabolism remained when the potential effect of body mass was taken into account as a covariate (RMRdry summer = 19.9 ± 7.3 ml O_2_ min^−1^ kg^−1^ and winter = 12.2 ± 6.8 ml O_2_ min^−1^ kg^−1^; LRT = 11.57, *P* < 0.001; Fig. 2C).

Similar seasonal results were found for metabolic rate while resting in 4°C water. Initial (pre-fast) absolute metabolic rate in water (RMRwet) was significantly greater in the summer (267.9 ± 56.8 ml O_2_ min^−1^) compared with the winter (193.4 ± 58.5; LRT = 14.66, *P* < 0.001; Fig. 2B). Again, adding body mass as a model covariate did not change the significance of these seasonal differences (RMRwet summer = 20.2 ± 4.4 ml O_2_ min^−1^ kg^−1^ and RMRwet winter = 14.7 ± 4.5 ml O_2_ min^−1^ kg^−1^; LRT = 15.13, *P* < 0.001; Fig. 2D).

The testing environment (wet or dry) was not a significant factor in predicting pre-fast metabolism, such that average pre-fasting metabolism was not different between measurements made in ambient air and 4°C water (RMRdry = 205.3 ± 104.6 ml O_2_ min^−1^ and RMRwet = 226.1 ± 68.2 ml O_2_ min^−1^; LRT = 2.89, *P* = 0.09). Neither body mass nor season was a significant model factor, meaning that the lack of difference attributable to testing environment for pre-fast metabolic rates held true regardless of season and when the potential effect of body mass was accounted for (RMRdry = 15.6 ± 7.9 ml O_2_ h^−1^ kg^−1^ and RMRwet = 17.1 ± 5.2 ml O_2_ min^−1^ kg^−1^; LRT = 1.21, *P* = 0.27).

### Metabolic changes due to fasting

Overall, there were no significant changes in resting metabolic rates (RMRdry pre- vs. post-fast) over the course of the fasts, such that pre-fast metabolic rate measured in ambient air was not different from post-fast metabolic rate (LRT = 0.11, *P* = 0.74; Fig. 1D). However, there was a significant seasonal effect observed (LRT = 15.13, *P* < 0.001), indicative of differences in the nature of the response between seasons. A season-specific examination of the data confirmed no statistically significant changes in average resting metabolic rate (RMRdry) during either the winter or the summer trials (winter change from 161.4 ± 94.0 to 160.3 ± 74.6 ml O_2_ min^−1^, LRT = 0.003, *P* = 0.95; summer from 261.3 ± 91.5 to 213.7 ± 95.9 ml O_2_ min^−1^, LRT = 2.38, *P* = 0.12; Fig. 2A). This lack of change in resting metabolism in air over the course of the fasts in either the winter or the summer trials held true when body mass was taken into account (winter change from 12.2 ± 6.8 to 12.9 ± 6.0 ml O_2_ min^−1^ kg^−1^, LRT = 0.10, *P* = 0.75; summer from 19.9 ± 7.3 to 17.5 ± 8.0 ml O_2_ min^−1^ kg^−1^, LRT = 2.32, *P* = 0.13; Fig. 2C).

Overall, there was a significant decrease between pre- and post-fast measures of metabolism measured in 4°C water (RMRwet 226.1 ± 68.2 vs. 195.7 ± 49.3 ml O_2_ min^−1^; LRT = 5.30, *P* = 0.02). However, the statistical model that included season to explain changes in wet metabolic rate was significantly stronger than the null (pre- vs. post-fast only) model, suggesting a differential seasonal response. Analysed by season, there was no significant decrease in metabolism in water over the fast during the winter trials (from 193.4 ± 58.5 to 167.6 ± 36.7 ml O_2_ min^−1^; *P* = 0.055) but there was a significant decrease in the summer trials (from 267.9 ± 56.8 to 231.7 ± 39.1 ml O_2_ min^−1^; *P* = 0.01; Fig. 2B).

Of course, part of the apparent seasonal effect of changes in metabolism in cold water in response to fasting may be related to seasonal differences in body mass loss. For the winter trials, neither trial (pre- vs. post-fast) nor body mass was a significant model factor, confirming no significant changes in RMRwet over the fasts (from 14.7 ± 4.5 to 13.4 ± 2.9 ml O_2_ min^−1^ kg^−1^; LRT = 2.12, *P* = 0.15; Fig. 2D). For the summer trials, trial was a significant fixed effect and body mass was a significant covariate, indicating that, while at least a portion of the observed difference in pre- vs. post-fast metabolism in 4°C water was attributable to changes in body mass, there remained a significant fasting effect even when this was controlled for (from 20.2 ± 4.4 to 18.8 ± 3.7 ml O_2_ min^−1^ kg^−1^; LRT = 5.1, *P* = 0.02).

### Factors affecting rates of body mass loss

Given that both rates of mass loss during the fast and pre-fast metabolic rates were greater on average in the summer than the winter, we examined whether there was a significant linear relationship between the observed rates of mass loss and initial metabolic rates. The linear relationship between mass loss (in kilograms) over the course of the fast and initial RMR in ambient air varied significantly by season (LRT = 18.6, *P* < 0.001). When each season was examined separately, there was still a linear relationship for winter, wherein greater pre-fast metabolic rates resulted in greater rates of mass loss during the fast. However, there was no significant relationship between pre-fast metabolic rate and rate of mass loss over the fast during the summer trials (i.e. slope was not significantly different from zero).

In a similar manner, we examined whether the amount of food that the fur seal was ‘deprived of’ during the fasting period (average daily intake based upon 7 days prior to the fast, in kilograms per day) was a predictor of the amount of mass lost during the fast. Examining data from both the winter and summer trials together, food intake prior to the fast was able to predict rates of mass loss (in kilograms per day; LRT = 11.4, *P* < 0.001), but season was also a significant factor. When each season was examined separately, the change in mass could be predicted from food intake during the winter trials (ANOVA *F*-test intercept, *P* = 0.001 and slope, *P* = 0.01). Specifically, in the winter months, animals consuming greater amounts of food prior to the fasts experienced greater rates of mass loss over the course of the fast. However, there was no significant relationship between previous levels of food intake and changes in body mass over the fast during the summer trials.

## Discussion

Metabolic depression is a common physiological response to normal or unexpected periods of nutritional deprivation, typically measured as a decrease in resting metabolic rate (Guppy and Withers, 1999). Traditionally, the onset of metabolic depression is thought to be associated biochemically with the shift from glycogenolysis to gluconeogenesis (phase 2 fasting; Cahill, 1978; Castellini and Rea, 1992; Cherel *et al.*, 1992). However, changes in metabolism in response to fasting are not universal, and the tendency to implement this physiological change may depend upon factors related to ontogeny, life history, season or the extent and nature of the food restriction.

We predicted that the young northern fur seals in our study would exhibit metabolic depression in response to short-term acute fasts, based on the reported response of the young of other otariid species. For example, 7- to 10-week-old wild Antarctic fur seals (*Arctocephalus gazelle*) pups demonstrated a 13% decrease in resting metabolism (Arnould *et al.*, 2001) after 4 days of fasting. Captive Steller sea lion pups (6–14 weeks old) demonstrated significant (∼7%) decreases in metabolism in response to fasts identical to those implemented in the present study (D. A. S. Rosen and A. W. Trites, unpublublished data). In contrast, the fur seals in our study demonstrated no significant changes in resting metabolic rate over the course of the 65 h fasts.

One important ecological difference between the animals in the different metabolic studies is that the Antarctic fur seals and Steller sea lions were developmentally immature in comparison to the fur seals in our study, in that they would still have been nursing in the wild (in the laboratory study with the Steller sea lions, they were still consuming milk formula). The northern fur seals, in contrast, were already weaned (at ∼4 months of age) at the start of the study and were at a stage when they would have begun foraging on their own. Likewise, subantarctic fur seal pups (*Arctocephalus tropicalis*) exhibited a 30% decrease in metabolism within 4 days of fasting (Verrier *et al.*, 2009). While these animals were older (∼7 months) than the fur seals in our study, they were also still dependent on their mothers and were subject to prolonged periods of fasting at this stage of their natural history due to maternal foraging trips. In other words, the difference observed in the metabolic response is likely to be attributable to the fact that the fast was considered ‘unpredicted’ within the context of the life history of the northern fur seals, whereas most other studies of young otariids have been conducted at life-history stages where periodic fasting episodes are the norm (e.g. Arnould *et al.*, 2001; Beauplet *et al.*, 2003; Verrier *et al.*, 2009).

Theoretically, metabolic depression is implemented during episodes of under-nutrition to minimize energetic expenditures and decrease rates of mass loss. Given the lack of physiological adjustment apparent in the resting metabolism of the fur seals, it is not surprising that there were significant consequences to this short episode of food deprivation. In addition, given the seasonal nature of the northern fur seals' energy budget (Rosen and Trites, 2010), it was not surprising that these consequences differed by season, with our fur seals losing significantly more mass during the summer months than the winter months. However, the question remains regarding the proximate cause of the observed seasonal differences in rates of mass loss during the fast.

Conceivably, a proportion of the observed seasonal differences in rates of mass loss could have been a byproduct of different sources of tissue catabolism. Blubber has a higher energy concentration per gram than lean tissues, largely reflective of the energetic differences between lipid and protein constituents (Schmidt-Nielsen, 1997). Therefore, the lower rates of mass loss in the summer could, in theory, have been due to the preferential catabolism of lipid vs. protein compared with the winter. This could be a bioenergetically wise decision, in that the fur seals would be less likely to require the additional insulative component in the wild during the summer. However, this strategy seems unlikely given the fur seals' lower initial lipid content in the summer and their apparent lack of post-fast thermal response in cold water.

We believe that the higher rates of mass loss exhibited in the summer months were due to the higher metabolic costs associated with this time of year. This was specifically demonstrated by the higher rates of resting metabolism during the summer. Given that resting metabolism did not change over the course of fasting, it represents a source of immutable energetic expenditure that must be met by either food intake (not possible during fasting) or tissue catabolism (apparent as differences in rates of mass loss). This higher RMR during the summer trials is likely to be a consequence of the fur seals' reliance on this period for lean tissue growth (Trites and Bigg, 1996; Rosen *et al.*, 2012). These increased demands of higher resting metabolic rates and growth were mirrored by a significantly greater rate of energy consumption in the summer compared with the winter trials. In fact, up-regulation of digestive capacity is not only a strong contributor to higher rates of resting metabolism during the summer months, but may also preclude short-term down-regulation in response to unexpected food shortages.

The strategy to prioritize lean tissue growth in the summer months may also explain why there was a stronger relationship between resting metabolism and rate of mass loss within the winter trials than the summer ones. It has been demonstrated that food-restricted Steller sea lions will actually increase core mass while losing overall body mass during critical times of the year (Rosen and Trites, 2005), thereby confounding the relationship between changes in body mass and changes in energy storage during the summer growth period.

In addition to the direct loss of body mass, there was also potential for a further seasonal effect of food deprivation due to the loss of body lipid (even assuming that the proportion of lipid loss was equal between seasons). In pinnipeds, the hypodermal lipid (blubber) layer generally serves both as an energy reserve and as insulation. Therefore, loss of lipid mass can cause an increase in thermoregulatory costs due to decreasing insulation, which potentially serves to increase the energy debt further and result in greater mass loss, leading to a downward spiral of mounting energy debt (Rosen *et al.*, 2007).

We predicted that the mass loss ensuing from the fast would increase the metabolic cost of being in cold water. In addition, we predicted that the thermal effect would have the greatest potential impact following fasts in the summer months, a time of the year when the fur seals are least likely to encounter cold waters in the wild, had lower absolute and relative initial lipid stores, and lost a greater proportion of body mass (presumably with concurrent loss of lipid reserves). This prediction was supported by the observation that larger (169–179 kg), older Steller sea lions exhibited a significant increase in metabolism following a similar fast when exposed to water at 2°C (D. A. S. Rosen, unpublished data). However, contrary to predictions, there was a complete lack of increased thermal response while in cold water following the fasting period.

The lack of thermal response while in water occurred despite the fact that a concurrent study of the same animals confirmed that the testing conditions were on the edge of their thermal neutral zone (Rosen and Trites, 2014). Presumably, the lack of response in the fur seals (even after presumed lipid loss during the fast) reflects a greater reliance on the insulative capacity of their pelage rather than their rather meagre (by comparison) hypodermal blubber (Liwanag *et al.*, 2012). However, it should be noted that, while lipid mass loss due to short-term food restriction may not have additional thermal consequences, the higher concurrent rates of protein loss during the summer may also have consequences for health and survival.

### Ecological implications

Little is currently known about the physiology of northern fur seals in the wild owing to their absence from land when 4–24 months of age. Our observations of fur seals in captivity, therefore, have several implications for how unpredicted changes in food supplies might affect northern fur seals in the wild during this cryptic period of their life history. Contrary to initial predictions, the fur seals did not exhibit significant metabolic depression in either winter or summer periods. This may have been because the period of imposed fasting (or, more probably, the realized mass loss; Øritsland, 1990) was insufficient to trigger such a response. However, this is unlikely given that significant decreases in metabolism have been observed in response to similar episodes of short-term fasting in other young otariids.

We suggest that the lack of metabolic response (or delay in implementing metabolic depression) is most likely to be related to the fact that northern fur seals would not usually be subject to longer periods of food deprivation at this age, and so would not be expected to have evolved a rapid metabolic response to short-term periods of food restriction. While a lack of metabolic adjustment will result in greater rates of mass loss during the period of fasting, these impacts may be offset by avoiding the bioenergetic and physiological consequences of metabolic down-regulation, such as the potential impacts on growth, thermoregulation and the ability to make efficient use of food resources when they become available (Storey and Storey, 2004).

However, the lack of metabolic adjustments and the inherent seasonal differences in metabolism suggest that short-term disruptions in the food supply would most dramatically affect northern fur seals in the summer season by imposing greater rates of mass loss.

Other studies with captive fur seals have previously suggested that the capacity of fur seals to overcome periods of food shortages by increasing subsequent levels of food intake are severely limited by innate digestive limitations (Rosen *et al.*, 2012). This is most likely to occur during the summer months, when the potential food deficit is highest and its impact on growth rates most pronounced. Therefore, future conservation efforts should concentrate on the availability of adequate prey for young northern fur seals in the North Pacific Ocean during the relatively brief, but vital period of summer growth.

## References

[1] Arnould JPY Boyd IL Speakman JR (1996) Measuring the body composition of Antarctic fur seals (*Arctocephalus gazella*): validation of hydrogen isotope dilution. Physiol Zool 69: 93–116.

[2] Arnould JPY Green JA Rawlins DR (2001) Fasting metabolism in Antarctic fur seal (*Arctocephalus gazella*) pups. Comp Biochem Physiol A Mol Integr Physiol 129: 829–841. 11440869 10.1016/s1095-6433(01)00339-7

[3] Baker JD Donohue MJ (2000) Ontogeny of swimming and diving in northern fur seal (*Callorhinus ursinus*) pups. Can J Zool 78: 100–109.

[4] Beauplet G Guinet C Arnould JPY (2003) Body composition changes, metabolic fuel use, and energy expenditure during extended fasting in subantarctic fur seal (*Arctocephalus tropicalis*) pups at Amsterdam Island. Physiol Biochem Zool 76: 262–270. 12794680 10.1086/367951

[5] Cahill GF (1978) Famine symposium – physiology of acute starvation in man. Ecol Food Nutr 6: 221–230.

[6] Castellini MA Rea LD (1992) The biochemistry of natural fasting at its limits. Experientia 48: 575–582. 1612138 10.1007/BF01920242

[7] Cherel Y Heitz A Calgari C Robin J-P Le Maho Y (1992) Relationships between lipid availability and protein utilization during prolonged fasting. J Comp Physiol B 162: 305–313. 1506487 10.1007/BF00260757

[8] Dalton AJM Rosen DAS Trites AW (2014) Broad thermal capacity facilitates the primarily pelagic existence of northern fur seals (*Callorhinus ursinus*). Mar Mammal Sci (in press) doi:10.1111/mms.12103.

[9] Donohue MJ Costa DP Goebel ME Baker JD (2000) The ontogeny of metabolic rate and thermoregulatory capabilities of northern fur seal, *Callorhinus ursinus*, pups in air and water. J Exp Biol 203: 1003–1016. 10683160 10.1242/jeb.203.6.1003

[10] Glazier DS (2005) Beyond the ‘3/4-power law’: variation in the intra- and interspecific scaling of metabolic rate in animals. Biol Rev 80: 611–662. 16221332 10.1017/S1464793105006834

[11] Guppy M Withers P (1999) Metabolic depression in animals: physiological perspectives and biochemical generalizations. Biol Rev 74: 1–40. 10396183 10.1017/s0006323198005258

[12] Heusner AA (1982) Energy metabolism and body size. 1. Is the 0.75 mass exponent of Kleiber's equation a statistical artifact? Respir Physiol 48: 1–12. 7111915 10.1016/0034-5687(82)90046-9

[13] Hoopes LA (2007) Metabolic and thermoregulatory capabilities of juvenile Steller sea lions (*Eumetopias jubatus*). PhD thesis. Texas A&M University, College Station, TX.

[14] Houser DS Costa D (2003) Entrance into stage III fasting by starveling northern elephant seal pups. Mar Mammal Sci 19: 186–197.

[15] Keys A Brozek A Henschel A Micckelsen O Taylor HL (1950) The Biology of Human Starvation. University of Minnesota Press, Minneapolis, MN.

[16] Lander RH (1982) A life table and biomass estimate for Alaskan fur seals. Fish Res 1: 55–70.

[17] Liwanag HEM Berta A Costa DP Budge SM Williams TM (2012) Morphological and thermal properties of mammalian insulation: the evolutionary transition to blubber in pinnipeds. Biol J Linnean Soc 107: 774–787.

[18] Lydersen C Kovacs KM Hammill MO (1997) Energetics during nursing and early postweaning fasting in hooded seal (*Cystophora cristata*) pups from the Gulf of St Lawrence, Canada. J Comp Physiol B 167: 81–88. 9120069 10.1007/s003600050050

[19] National Marine Fisheries Service (2007) Conservation Plan for the Eastern Pacific Stock of Northern Fur Seal (*Callorhinus ursinus*). National Marine Fisheries Service, Juneau, AK, 100 pp.

[20] Nordøy ES Ingebretsen OC Blix AS (1990) Depressed metabolism and low protein catabolism in fasting gray seal pups. Acta Physiol Scand 139: 361–369. 2368622 10.1111/j.1748-1716.1990.tb08935.x

[21] Nordøy ES Aakvaag A Larsen TS (1993) Metabolic adaptations to fasting in harp seal pups. Physiol Zool 66: 926–945.

[22] Øritsland NA (1990) Starvation survival and body composition in mammals with particular reference to *Homo sapiens*. Bull Math Biol 52: 643–655. 2224284 10.1016/s0092-8240(05)80371-4

[23] Packard GC Birchard GF (2008) Traditional allometric analysis fails to provide a valid predictive model for mammalian metabolic rates. J Exp Biol 211: 3581–3587. 18978222 10.1242/jeb.023317

[24] Pinheiro JC Bates DM (2000) Mixed Effects Models in S and S-PLUS. Springer Verlag, New York.

[25] Ragen TJ Antonelis GA Kiyota M (1995) Early migration of northern fur seal pups from St. Paul Island, Alaska. J Mammal 76: 1137–1148.

[26] Rea LD Costa DP (1992) Changes in standard metabolism during long-term fasting in northern elephant seal pups (*Mirounga angustirostris*). Physiol Zool 65: 97–111.

[27] Rea LD Rosen DAS Trites AW (2000) Metabolic response to fasting in 6-week-old Steller sea lion pups (*Eumetopias jubatus*). Can J Zool 78: 890–894.

[28] Reilly JJ (1991) Adaptations to prolonged fasting in free-living weaned gray seal pups. Am J Physiol 260: R267–R272. 1996712 10.1152/ajpregu.1991.260.2.R267

[29] Reilly JJ Fedak MA (1990) Measurement of the body composition of living gray seals by hydrogen isotope dilution. J Appl Physiol 69: 885–891. 2246176 10.1152/jappl.1990.69.3.885

[30] Rosen DAS Trites AW (2002) Changes in metabolism in response to fasting and food restriction in the Steller sea lion. Comp Biochem Physiol B 132: 389–399. 12031465 10.1016/s1096-4959(02)00048-9

[31] Rosen DAS Trites AW (2005) Examining the potential for nutritional stress in Steller sea lions: physiological effects of prey composition. J Comp Physiol B 175: 265–273. 15900507 10.1007/s00360-005-0481-5

[32] Rosen DAS Trites AW (2010) Split personalities: seasonal energetic priorities in young northern fur seals. Proceedings of the Comparative Nutrition Society, 5–11 August 2010, Tucson, AZ, pp 178–181.

[33] Rosen DAS Trites AW (2014) Thermal limits in young northern fur seals, Callorhinus ursinus. Mar Mammal Sci (in press) doi:10.1111/mms.12097.

[34] Rosen DAS Winship AJ Hoopes LA (2007) Thermal and digestive constraints to foraging behaviour in marine mammals. Phil Trans R Soc London B 362: 2151–2168. 17472918 10.1098/rstb.2007.2108 PMC2442860

[35] Rosen DAS Young BL Trites AW (2012) Rates of maximum food intake in young northern fur seals (*Callorhinus ursinus*) and the seasonal effects of food intake on body growth. Can J Zool 90: 61–69.

[36] Schmidt-Nielsen K (1997) Animal Physiology: Adaptation and Environment. Cambridge University Press, Cambridge.

[37] Shero M Andrews R Lestyk K Burns J (2012) Development of the aerobic dive limit and muscular efficiency in northern fur seals (*Callorhinus ursinus*). J Comp Physiol B 182: 425–436. 22001970 10.1007/s00360-011-0619-6

[38] Storey KB Storey JM (2004) Metabolic rate depression in animals: transcriptional and translational controls. Biol Rev 79: 207–233. 15005178 10.1017/s1464793103006195

[39] Trites AW (1989) Estimating the juvenile survival rate of male northern fur seals (*Callorhinus ursinus*). Can J Fish Aquat Sci 46: 1428–1436.

[40] Trites AW (1992) Northern fur seals: why have they declined? Aquat Mamm 18: 3–18.

[41] Trites AW Bigg MA (1996) Physical growth of northern fur seals (*Callorhinus ursinus*): seasonal fluctuations and migratory influences. J Zool London 238: 459–482.

[42] Verrier D Groscolas R Guinet C Arnould JPY (2009) Physiological response to extreme fasting in subantarctic fur seal (*Arctocephalus tropicalis*) pups: metabolic rates, energy reserve utilization, and water fluxes. Am J Physiol Regul Integr Comp Physiol 297: R1582–R1592. 19776248 10.1152/ajpregu.90857.2008

[43] Wilson PN Osbourn DF (1960) Compensatory growth after undernutrition in mammals and birds. Biol Rev 35: 324–363. 13785698 10.1111/j.1469-185x.1960.tb01327.x

[44] Withers PC (1977) Measurement of VO_2_, VCO_2_, and evaporative water loss with a flow-through mask. J Appl Physiol Respir Environ Exerc Physiol 42: 120–123. 833070 10.1152/jappl.1977.42.1.120

[45] Worthy GAJ Lavigne DM (1987) Mass loss, metabolic rate, and energy utilization by harp and gray seal pups during the postweaning fast. Physiol Zool 60: 352–364.

[46] York AE (1994) The population dynamics of Northern sea lions, 1975–1985. Mar Mammal Sci 10: 38–51.

